# Early interventions in risk groups for schizophrenia: what are we waiting for?

**DOI:** 10.1038/npjschz.2016.3

**Published:** 2016-03-09

**Authors:** Iris E Sommer, Carrie E Bearden, Edwin van Dellen, Elemi J Breetvelt, Sasja N Duijff, Kim Maijer, Therese van Amelsvoort, Lieuwe de Haan, Raquel E Gur, Celso Arango, Covadonga M Díaz-Caneja, Christiaan H Vinkers, Jacob AS Vorstman

**Affiliations:** 1 Department of Psychiatry, Brain Center Rudolf Magnus, University Medical Center Utrecht, Utrecht, the Netherlands; 2 Semel Institute for Neuroscience and Human Behavior, Departments of Psychiatry and Biobehavioral Sciences and Psychology, University of California, Los Angeles, CA, USA; 3 Department of Psychiatry and Psychology, Maastricht University, Maastricht, The Netherlands; 4 Department of Psychiatry, Academic Psychiatric Centre, AMC, Amsterdam, The Netherlands; 5 Department of Psychiatry, Perelman School of Medicine, University of Pennsylvania, Philadelphia, PA, USA; 6 Child and Adolescent Psychiatry Department, Hospital General Universitario Gregorio Marañón, Instituto de Investigación Sanitaria Gregorio Marañón, IiSGM, Centro de Investigación Biomédica en Red de Salud Mental, CIBERSAM, School of Medicine, Universidad Complutense, Madrid, Spain

## Abstract

Intervention strategies in adolescents at ultra high-risk (UHR) for psychosis are promising for reducing conversion to overt illness, but have only limited impact on functional outcome. Recent studies suggest that cognition does not further decline during the UHR stage. As social and cognitive impairments typically develop before the first psychotic episode and even years before the UHR stage, prevention should also start much earlier in the groups at risk for schizophrenia and other psychiatric disorders. Early intervention strategies could aim to improve stress resilience, optimize brain maturation, and prevent or alleviate adverse environmental circumstances. These strategies should urgently be tested for efficacy: the prevalence of ~1% implies that yearly ~22 in every 100,000 people develop overt symptoms of this illness, despite the fact that for many of them—e.g., children with an affected first-degree family member or carriers of specific genetic variants—increased risk was already identifiable early in life. Our current ability to recognize several risk groups at an early age not only provides an opportunity, but also implies a clinical imperative to act. Time is pressing to investigate preventive interventions in high-risk children to mitigate or prevent the development of schizophrenia and related psychiatric disorders.

## Introduction

### Current treatment of schizophrenia starts too late

Schizophrenia is a complex brain disorder with a heterogeneous presentation and variable outcome. Schizophrenia is relatively common, with a prevalence ~1%, depending on gender, country, and degree of urbanicity.^1^ A substantial proportion of patients with schizophrenia experience marked impairments in multiple domains necessary for daily functioning, affecting their ability to maintain social relationships, sustain employment, and live independently. In addition, the economic burden is substantial: In Europe, the cost of schizophrenia-spectrum disorders, including both direct and indirect expenses, was estimated to be almost 94 billion € in 2010.^2^ Remission of psychotic symptoms can be achieved for the majority of patients,^3,4^ but social and professional impairments generally persist after remission from psychosis.^5,6^ The reason is that functional outcome is strongly associated with the presence and severity of cognitive and negative symptoms,^7^ indicating that positive symptoms (i.e., hallucinations and delusions) are not the core symptoms of the illness. Although modest improvements in cognitive and social functioning are achievable in adult patients,^8^ severe deficits in these domains are hard to overcome. To increase therapeutic impact, we should therefore aim to prevent the development of severe social and cognitive impairments before they are established.

### Developmental and cognitive abnormalities early in the trajectory of schizophrenia

Overt psychosis is not the beginning, nor the core feature of schizophrenia, and should consequently not be the main target for early intervention and prevention. During the past two decades, research has focused on the period directly preceding the first psychotic episode when subclinical psychotic features emerge. These ultra high-risk (UHR) studies consistently observe widespread deficits across multiple cognitive domains,^9^ as well as reduced social abilities^10^ in youth with attenuated psychotic symptoms. To prevent psychosis in this UHR stage, interventions with psychotherapy and nutritional supplements^11,12^ have been applied with some success. However, current data suggest that the cognitive deficits observed at baseline in UHR individuals do not improve with such interventions and usually remain constant when progressing from the UHR stage to psychosis.^13^ in fact, most people who meet UHR criteria already have a major psychiatric disorder and the majority of those who do not transit to a psychotic disorder still end up with significant psychopathology and/or social disability.^14^ Indeed, most of the variance in functional outcome is predicted by neurocognitive decrements already present at the start of the UHR phase, regardless of transition to overt psychosis.^15,16^

A logical inference from these important observations is that strategies to prevent social-cognitive deficits should be applied earlier in the trajectory, i.e., during childhood and early adolescence, or even pre- or perinatally.^17^ Several epidemiologic studies demonstrate that children who developed schizophrenia as adults have, on average, a significantly lower IQ at age 4 and 7 years,^18^ compared with normally developing children. These early childhood cognitive deficits continue to progress at the onset of adolescence, with reduced cognitive functioning at age 12 years,^19^ as well as significantly lower school performance at 13 and 14 years.^20^ Importantly, a meta-analysis on IQ during the course of schizophrenia indicates further cognitive decline occurring before the onset of adolescence.^21^ This was confirmed in a longitudinal birth cohort with a lengthy follow-up period observing that children, subsequently diagnosed with schizophrenia in adulthood, had a cognitive decline of 9 IQ points at age 13 and a decline of 15 IQ points around the time of diagnosis as compared with peers who did not develop the disorder.^22^ Recently, findings in a large prospective longitudinal cohort of patients at increased risk for schizophrenia due to the 22q11.2 deletion showed a similar decrease in IQ, preceding the first psychotic episode by 7 years on average.^23^ Children who develop schizophrenia may face other problems, e.g., with motor coordination and behavior.^24^

From these observations, we can infer that several different neurodevelopmental pathways may lead to schizophrenia, while similar neurodevelopmental deviations may lead to different psychiatric disorders. Therefore, preventive interventions for schizophrenia are likely to target different risk groups and may decrease risk not only for schizophrenia and related psychotic disorders, but also for a broader range of mental disorders, including affective, personality, and substance abuse disorders.^25^

The goal of this paper is to discuss early interventions that may have the potential to improve outcome by safeguarding cognitive and social development (primary goal) or by preventing the full-blown manifestation of psychosis (secondary goal). We will also outline which specific subgroups within the population may be most amenable to such strategies and, finally, what ethical and economic aspects are relevant in relation to the use of very early prevention strategies.

## How to intervene: potential strategies for prevention in at-risk groups

First, we propose strategies that can improve suboptimal maturation of neuronal pathways during childhood. Second, we examine interventions that can reduce environmental insults or mitigate their impact. Third, we explore strategies that can improve resilience, even in the presence of negative circumstances and/or genetic risk factors.

### Improving suboptimal maturation of neuronal pathways

While abnormal dopaminergic signaling is strongly associated with the onset of psychosis,^26^ gamma-aminobutyric acid (GABA)-ergic and glutamatergic signaling defects may be critically involved in the development of social and cognitive deficits in schizophrenia.^27–29^ While GABA is an inhibitory neurotransmitter in adult life, it is excitatory in early fetal brain development. The chloride transporter KCC2 switches GABA from excitatory to inhibitory. This chloride transporter can be stimulated by activation of postsynaptic α7-nicotinic acetylcholine receptors.^30^

#### Glutamatergic compounds

To date, several strategies to alter glutamatergic neurotransmission have been investigated for their effect on cognition in patients with schizophrenia using glycine-site NMDA-modulating compounds such as glycine, d-serine, d-cycloserine, and the glycine transporter 1 (GlyT1) inhibitor sarcosin.^31^ The evidence with regard to the addition of the NMDA receptor partial agonist d-cycloserine to antipsychotic therapy is mixed, with either exacerbation or alleviation of positive symptoms^32^ and amelioration of negative symptoms.^33^ Efficacy of d-serine treatment with regard to cognitive symptoms in schizophrenia has not been convincingly demonstrated.^34,35^ In addition, a recent meta-analysis demonstrated that glutamate-positive modulators are not beneficial in the treatment of cognitive symptoms in schizophrenia.^36^ Hence, studies thus far have not shown efficacy of these glutamate-modifying compounds in adult patients with schizophrenia. Yet, this approach merits further exploration, in particular the use of such agents during the early phases of schizophrenia. If applied early, when abnormalities in glutamatergic transmission start to emerge in those who develop schizophrenia, their efficacy may be increased. Indeed, a recent preclinical study showed far-reaching normalizing effects of d-serine on brain function in *Pick1* knockout mice, provided it was applied early. The behavioral deficits associated with loss of function of *Pick1* could be reversed, but only when d-serine was administered neonatally, not during adult age.^37^ In addition, there may be other glutamatergic-modulating agents with potential. For instance, there is emerging evidence for a therapeutic effect of pregnenolone in patients with schizophrenia, a molecule with both neurosteroid and NMDA receptor-modulating effects.^38^ More clinical studies are required to examine effects and possible adverse effects of these and other glutamate-modifying drugs, while pre-clinical studies are needed to elucidate the mechanisms of action of these molecules on deviating neurodevelopmental pathways and the optimal timing of such interventions.

#### GABA-ergic compounds

Another potential approach to enhancing cognition in those at risk for psychosis is by influencing GABAergic pathways. Two important classes of selective GABAergic drugs show promise: α_5_-selective inverse GABA_A_ receptor agonists and α_2/3_-selective GABA_A_ receptor agonists.^39^ The α_5_ subunit, which is predominantly expressed in the hippocampus, is essential in modulating the interneuron-pyramidal network. While the evidence for pro-cognitive effects of α_5_-selective inverse agonists is equivocal in humans,^40^ there is potential for modulation of GABA_A_ receptors with an α_5_ subunit.^41,42^ The results of a first study on pro-cognitive effects in adult patients with schizophrenia, using the α_2/3_-selective positive allosteric modulator MK0777, were disappointing.^42^ Nevertheless, the rationale for this strategy remains appealing given the large body of evidence for altered expression and functionality of GABA_A_ receptor α_2/3_ subunits in schizophrenia and their relevance for cognition.^39^ In particular, similar to the glutamate system, it is possible that GABAergic interventions are more effective when applied earlier in life, when the GABA system is still building up neural connections and therefore more amenable to change. In addition to modifying GABA_A_ receptor functionality, other possible approaches to modulate the GABA system should also be studied, including modulating GABA metabolism, using cation/chloride transporters, neurosteroids or transcranial magnetic stimulation.^43^ For instance, bumetanide, which inhibits the cation/chloride transporter NKKC1 and thereby indirectly modulates GABAergic transmission, has been shown to change cortical circuits and reverse sensorimotor-gating deficits.^44,45^ However, interference with the GABA_A_ receptors is not without danger, given the increased risk for triggering epilepsy. Therefore, the development of alternative approaches to modify GABAergic neurotransmission with milder side-effect profiles is needed.

#### Choline supplementation

Perinatal supplementation with choline is shown to improve cognitive function in animal models of schizophrenia.^46^ A trial in 100 human newborns showed that babies with perinatal supplementation more often showed adequate inhibition at the auditory P50 paradigm than infants without supplementation,^47^ indicating that a choline-enhancing diet for pregnant women with high risk or supplementation for neonates may have positive effects on cholinergic function and facilitate the development of adequate cortical inhibition.

#### Anti-oxidants and agents that affect the immune response

Findings from murine studies suggest that both glutamatergic and GABA-ergic transmission may be negatively affected by increased immune activation and a negative redox balance in the brain.^48^ A negative redox balance causes oxidative stress, and results from an abundance of reactive oxygen and nitrogen species relative to the availability of antioxidants. Both immune system and redox abnormalities have been observed in the peripheral blood of patients with schizophrenia, but also in those at increased risk.^49–52^ The most important antioxidant for the brain is glutathione, which plays a critical role in myelination and white matter maturation,^53^ and can be replenished by nutritional supplement of its amino acid precursor *N*-acetylcysteine (NAC). NAC is non-toxic, has few side effects^54^ and induces an upregulation of glutathione synthesis, which can neutralize extra production of oxygen and nitric radicals in a stressed brain.^48^ NAC also has mild anti-inflammatory effects, likely via its antioxidant properties.^54^ Several rodent studies demonstrate that restoration of the redox balance by NAC or other antioxidants during early developmental stages can mitigate adverse effects of stress on brain maturation,^55^ and rescue social or cognitive deficit phenotypes induced by early social isolation,^56^ or prevent the development of these phenotypes induced by neonatal hippocampal lesions.^57^ In addition to replenishing glutathione, NAC also plays a role in the regulation of synaptic NMDA signaling.^58^ Its positive effects on the redox balance, neuroinflammation, and NMDA receptor functioning in combination with its mild side-effect profile make NAC a preferred candidate molecule for further study with preventive potential when applied early in the course of schizophrenia.

Omega-3 type polyunsaturated fatty acids (PUFAs) also have some antioxidative capacity^59^ and mild anti-inflammatory effects on the brain.^60^ PUFAs are an important component of neuronal and glial cell membranes and could facilitate synaptogenesis. Like NAC, PUFAs have a very mild side-effect profile, and thus would be a good candidate to study preventive effects when used early in development. One study reported significant positive effects of early administration of PUFAs compared with placebo in youth at risk for psychosis on both the transition rate to full-blown psychosis and broader functional outcome measures.^61^ Currently, recruitment for a new study is ongoing to replicate this finding.^62^ PUFAs were shown to have some beneficial effects on neurodevelopment and could mitigate the risk for childhood psychiatric disorders such as autism and ADHD.^63^ As all neurodevelopmental disorders form risk factors for schizophrenia,^64,65^ this could be an additional route to decrease the risk for schizophrenia in at-risk groups.

Another potential intervention to modulate the immune response with minimal side effects is the use of probiotics. The large impact of the microbiome on brain function is becoming increasingly clear. Several gut bacteria are capable of producing neurotransmitters such as GABA and acetylcholine.^66^ A “leaky gut” has been hypothesized to lead to increased inflammatory activation and have negative impact on brain maturation in several psychiatric disorders, including schizophrenia.^67^ Probiotics contain beneficial bacteria such as bifidobacterium and lactobacillus that can decrease systemic proinflammatory cytokines, increase neurotrophic factors, and reduce oxidative stress.^68^ In healthy adults, these agents have been shown to reduce anxiety and stress.^69^ Since probiotics have few side effects, this may be a promising intervention for children at increased risk to develop schizophrenia.

### Reducing environmental insults or their impact

The key biological system required for stress adaptation, the hypothalamic-pituitary-adrenal (HPA) axis, is abnormal in schizophrenia and altered HPA axis functionality has been related to both cognitive and negative symptoms in schizophrenia.^70–72^ Moreover, changes in the HPA axis may predate the onset of overt psychotic symptoms since UHR patients display a blunted cortisol stress response,^73^ altered basal cortisol levels,^74^ and abnormal pituitary volumes.^75^ In addition, the appeal of interventions aiming to reduce stress and improving resilience is based on strong evidence linking excessive stress with biological mechanisms associated with schizophrenia, including central nervous system immune activation,^76^ dopamine^77^ and glutamate^76^ release, and redox balance disruption.^78^ In addition to interventions directly aimed at reducing social stress, the prevention of drug abuse during adolescence may also be a relevant strategy given the bidirectional relation between drug abuse and stress. Not only are the exposure to excessive stressors and alterations in the HPA axis risk factors for drug abuse,^79^ the use of drugs can also be associated with a disruption of normal stress regulation.^80^

#### Social skills training to prevent bullying and social exclusion

Reducing bullying and peer rejection may improve social outcomes in the general population.^81^ Targeted anti-bullying programs therefore constitute a promising prevention strategy, as bullying is frequent among youth predisposed to schizophrenia or related disorders and often results in social isolation and chronic stress.^82,83^ The use of an individual coaching program for children and young adolescents can decrease the prevalence of bullying and improve social and cognitive functioning.^84^ Children who already have some delay in cognitive, social, or motor development are at even higher risk for being bullied and if that happens their risk for many different psychiatric disorders increases.^85^ A prospective study on >1,000 adolescents showed that the incidence of psychotic experiences decreased significantly in individuals whose exposure to bullying ceased over the course of the study,^86^ indicating that interventions that can stop bullying can impact the expression of psychosis vulnerability. These findings underscore the relevance of such programs in reducing stress from bullying and social isolation; the time has come therefore to examine their preventive potential when applied at an early age in children who are at increased risk for schizophrenia.

#### Early interventions to prevent drug abuse

In addition to its relation to stress regulation, drug abuse, especially when initiated in the early teens, has long been recognized as an important risk factor for schizophrenia.^87^ Although more studies are required for conclusive evidence,^88^ results thus far indicate that interventions for teens and their parents, which improve family communication and rule-setting, can reduce the rate of subsequent drug abuse and accompanying problem behavior.^88,89^ Given the serious consequences of early-drug use in youth at risk for schizophrenia, studying the efficacy of such programs in these populations is urgent.

### Improving resilience

#### Cognitive remediation

The main goal of cognitive remediation (CR) is to improve neuropsychological deficits, making it an appealing strategy to examine in the very early stages of schizophrenia in high-risk subjects, when cognitive deficits emerge. To date, there is increasing evidence for the benefits of CR applied after the first psychotic episode in patients with schizophrenia, with medium effect sizes on global cognition, which remain measurable after at least 1 year.^90^ Interestingly, these effects are larger when applied at younger age,^91^ after a shorter duration of the illness,^92^ and in individuals with higher cognitive levels.^93^ Benefits of CR applied to individuals in the UHR period with subclinical psychotic symptoms have been examined, indicating both cognitive and social gains.^94^ CR may be more effective when given in the period before the UHR stage when cognition can still be saved, i.e., during childhood.

#### Exercise training

Physical exercise may improve performance on different cognitive measures in patients with schizophrenia. Interestingly, exercise is associated with changes in gene expression related to brain plasticity,^95^ and improvements in both brain structure^96^ and connectivity.^97^ Although the majority of studies investigating the benefits of exercise have focused on the elderly, there is some evidence for similar efficacy in youth,^98^ making it an excellent intervention for primary or secondary prevention of cognitive decline and the development of severe psychiatric disorders.

## Who to target: potential candidates for early-preventive strategies

When the focus of potential intervention is shifted from the UHR phase to an earlier phase of development, more subjects will be exposed to interventions, thus necessitating an even more rigorous consideration of potential negative effects of at-risk designation and intervention strategies. Selected populations with high odds ratios to develop schizophrenia may provide a rational starting point for such studies, especially since even children from these risk groups who will not go on to develop schizophrenia may still be affected by cognitive and social impairments.

### Children with first-degree relatives with schizophrenia

Children with first-degree relatives suffering from schizophrenia-spectrum disorder have, on average, a 10-fold increased risk to develop the disease themselves.^99^ Cognition is also affected in adult first-degree relatives—even in those who have not themselves developed schizophrenia—albeit to a lesser degree compared with the impairments observed in clinically affected individuals.^9^ Risk for schizophrenia is not only increased in children from schizophrenia patients, but also in children from those with bipolar disorder.^100^ Interventions to prevent the development of cognitive deficits may therefore be relevant to those youngsters who are in fact early in the trajectory of schizophrenia, but also for the remainder of this group who will not develop the illness.

### Children with 22q11.2 deletion syndrome

**Figure 1:**
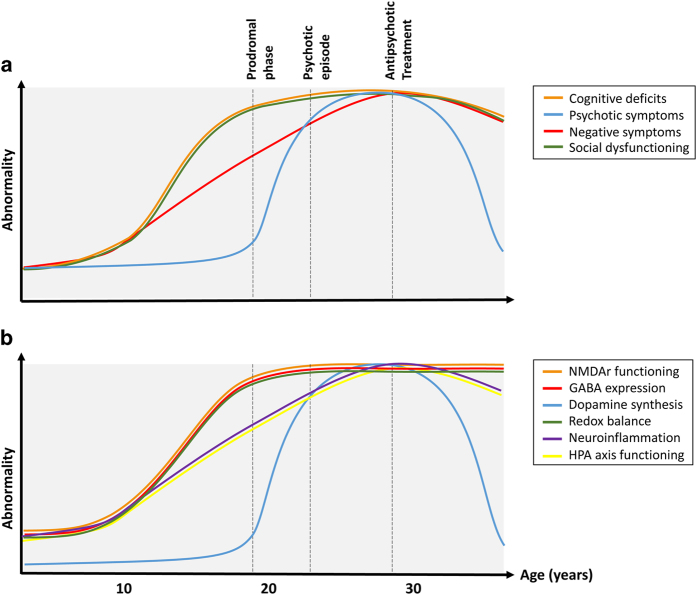
Hypothesized typical course of schizophrenia. (**a**) shows the clinical course of the disease. (**b**) shows the hypothesized course of the underlying molecular mechanisms.

The 22q11.2 deletion syndrome (22q11DS) has an estimated incidence of 1 in 2,000 and is the strongest single genetic risk factor for schizophrenia currently known, with approximately 1 in 4 individuals with 22q11DS developing the illness. In these individuals, the principal clinical characteristics cannot be distinguished from schizophrenia in the general population.^101^ Notably, a substantial proportion of 22q11DS children, when followed prospectively, display a decrease in IQ over time.^102^ Moreover, consistent with the observed cognitive abnormalities predating the onset of psychosis in the general population (see Figure 1),^19,20^ 22q11DS children who showed an early-cognitive decline had a threefold increased risk to be diagnosed later with a schizophrenia-spectrum disorder, compared with those without cognitive decline.^23^ The observation of increased plasma levels of the amino acid proline in approximately one third of individuals with 22q11DS^103^ is notable as evidence suggests that proline influences glutamatergic neurotransmission.^104,105^ Indeed, findings of several studies indicate that high plasma proline levels influence brain function in 22q11DS patients,^106,107^ suggesting that strategies to alter glutamatergic neurotransmission may be of particular relevance for this population. In conclusion, given their relative high-conversion rate and well-documented cognitive decline, children with a known 22q11 deletion are a small, but appealing target population for early-intervention strategies.

### Carriers of other CNVs associated with schizophrenia

In addition to 22q11DS, other copy number variants (CNVs) are also associated with increased risk of schizophrenia, including microdeletions at 1q21.1, 3q29, 15q11.2, and 17q12, as well as duplications at 15q11-13, 15q13.3, 16p11.2, and 16p13.1.^108^ Each of these variants is also associated with increased rates of other neurodevelopmental phenotypes, particularly autism spectrum disorders, intellectual disability, and epilepsy,^109^ as well as abnormal morphological features. These characteristics may cause this risk group to be vulnerable to social stress, therefore strategies to improve resilience and reduce social stress may be of particular benefit. These CNVs occur at very low rates in the population^108^ and even when considered together, they include only a small proportion of all schizophrenia patients. In contrast, the risk to develop schizophrenia in individual carriers of any one of these CNVs is substantial, with estimated odds ratios ranging between 2 and >30,^108^ making the population of carriers of these CNVs a target group for use of preventive measures early in the developmental trajectory.

### Youth experiencing transient psychotic symptoms

Psychotic experiences are common among young children, occurring in about 15% of children aged 9–11 years old.^110^ In most cases, these symptoms are transient, but approximately one third of this group experiences psychotic symptoms for over a year. For children with psychotic symptoms, the chance to develop a schizophrenia-spectrum disorder by the age of 26 is between 5 and 16 times increased, depending on the number and severity of the psychotic experiences.^111^

Children with psychotic experiences are not only at increased risk for schizophrenia, but also for other psychiatric disorders,^112^ and therefore general interventions to prevent deterioration may be of added value. Based on retrospective information obtained from adult patients with schizophrenia, 40% already experienced one or more psychotic features in childhood.^15^ Thus, preventive measures should be considered in children with psychotic experiences, in particular when they are persistent, when more than one feature is present, or when one severe psychotic symptom is present.

## Ethical and economic aspects of early-preventive strategies

With the exception of the pharmacological agents, the interventions discussed under 'How to intervene: potential strategies for prevention in at-risk groups' are non-invasive and safe, thereby fully respecting the adagium *primum non nocere* (i.e., first do no harm). Furthermore, some interventions, such as exercise training and bullying prevention programs are non-specific and likely to be beneficial for any youth, regardless the risk of developing schizophrenia. Nevertheless, the identification as a person at risk may have negative impact on a person’s well-being. Furthermore, without exception, the suggested interventions will be costly and it is therefore a key to provide the intervention for those who need it most.

### Disclosing presence of psychotic experiences and at-risk status

When communicating information about at-risk status for severe disorders to children and their families, a leading ethical question is whether telling children about their at-risk status harms them. This question should be considered in the context of the anticipated gain of the intervention, which is the prevention or mitigation of social and cognitive impairments and, ideally, of the full manifestation of a major psychiatric disorder. Communicating information about an increased risk for a major psychiatric disorder may cause anxiety, but it may also be beneficial for both the child and the family, as it may validate perceived social or cognitive problems or emerging psychotic experiences. Disclosure also provides the opportunity to educate child and family, for instance about other potential risk environments, early-symptom recognition, and lifestyle adjustments. More extensive discussions of considerations regarding ethical aspects of disclosing risk status have recently been published.^113,114^

### Economical aspects of early-intervention strategies

**Figure 2:**
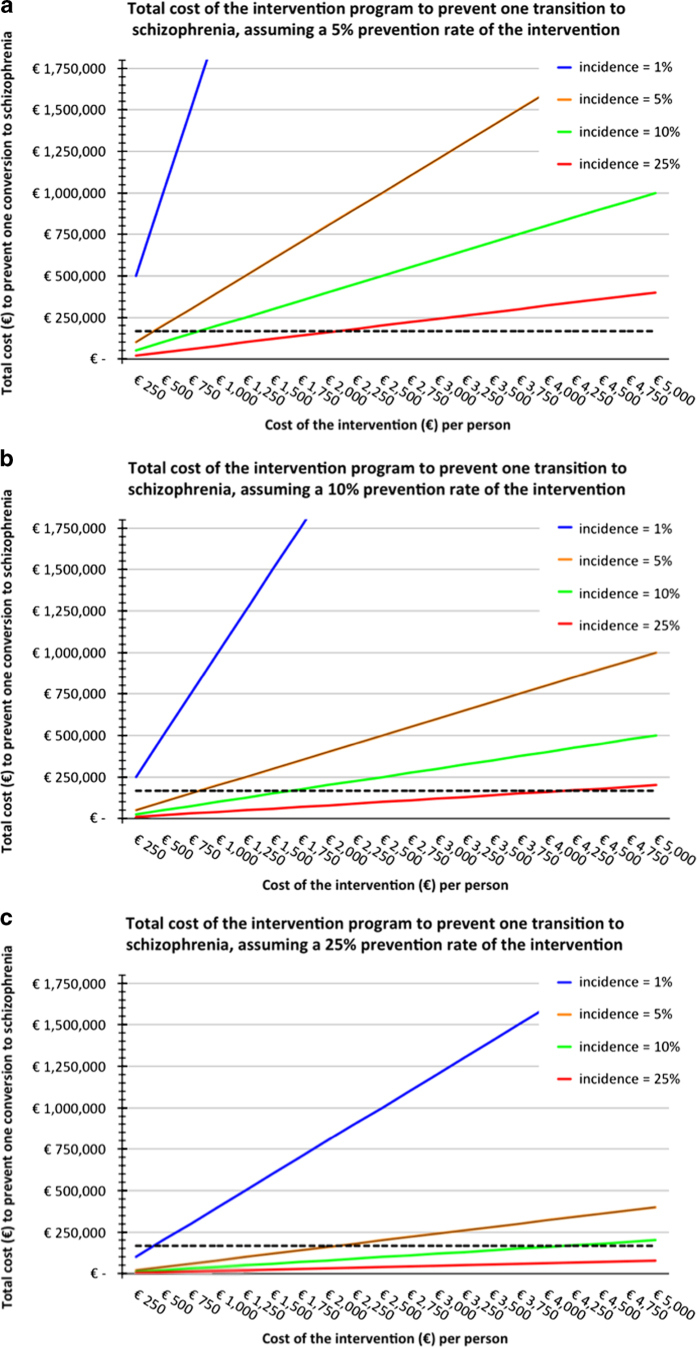
(**a**–**c**) Economical feasibility of early-intervention strategies. This figure indicates the total cost (in €) of any intervention strategy (*x* axis) to prevent the development of schizophrenia in one individual (*y* axis). (**a**–**c**) represent three scenarios based on different prevention rates of the intervention (5%, 10%, and 25% respectively). For reference, the black dotted line represents the estimated economic burden of one individual who develops a psychotic disorder calculated for a time span of 10 years. The area under the dotted line indicates an economic benefit since the costs of the preventive strategy to prevent one transition to schizophrenia outweighs the economic burden of one affected individual. Several factors influence whether a preventive intervention is cost-effective, including (i) the costs of the intervention (*x* axis). (ii) lifetime risk for a psychotic disorder in the target population (different line colors) with lower risks resulting in a reduced cost-effectiveness. The blue line refers to the risk for schizophrenia in the general population (1%), the red line applies to the selected population of 22q11DS individuals with a 25% lifetime incidence of schizophrenia and (iii) the effectiveness of the intervention; that is, in what proportion of individuals can the development of a psychotic disorder be averted as a result of the intervention?

Even when focusing on selected groups with high *a priori* risk for schizophrenia, it is important to assess the economical feasibility of early-intervention strategies. What are the expenses required for the implementation of the interventions and how do they weigh against the expected gains, i.e., the number of patients in whom a cognitive and social decline, and full development of schizophrenia is averted and improvement in cognitive and social functioning in those who do not make a transition to a psychotic disorder? In the absence of reliable data on effect sizes of the interventions discussed in this paper, we have modeled several scenarios to provide some insight into this question. In Figure 2, we present three scenarios (Figure 2a–c) based on different effect size assumptions, which are expressed as the prevention rates of the intervention (5%, 10%, and 25% respectively). For instance, a prevention rate of 10% implies that in 1 out of 10 individuals who are exposed to the intervention, the onset of social and cognitive dysfunction, and full development of schizophrenia is prevented. The general population, with a lifetime incidence of about 1%, is provided as a reference. The figure should be viewed while keeping in mind a conservative estimate of the economic burden related to one individual who develops a psychotic disorder, i.e., ~167k € per 10 years (indicated with the dotted black line).^115^ While interventions at low costs may be cost-effective in high-risk groups, we should also consider that there is a general willingness to pay for preventive interventions to safeguard future generations from severe dysfunction.

It is also important to realize that all the aforementioned risk groups have increased odds of multiple psychiatric disorders, not just schizophrenia. Therefore, early intervention in any of these groups may also be relevant in the prevention of a broad spectrum of psychiatric disorders, thereby enlarging the anticipated gain of the intervention. Finally, the selection of a high-risk population can be further improved when more expensive interventions are to be examined. This could be achieved by selecting those children who have more than one risk factor. For example, youth with subclinical psychotic symptoms and a family history of schizophrenia may be a strategic population to study such interventions before any associated cognitive dysfunction progresses to more severe levels.

## Discussion and conclusions

The past 20 years have largely increased our understanding of the developmental trajectory of schizophrenia. We now have some basic knowledge on environmental circumstances and neurobiological factors that can positively or negatively affect this developmental trajectory. Yet, this knowledge is not translated into preventive interventions in early stages. Each decade, many young people around the world develop schizophrenia without even a trend towards lowering the incidence. Time is pressing for research into early-preventive interventions to diminish the development of schizophrenia-spectrum disorders in future generations.

Psychosis is a core, but also a late symptom of schizophrenia, generally starting in early adolescence during the UHR period and progressing to the first psychotic episode in young adulthood. However, cognitive and social dysfunctions emerge at a much earlier stage and evolve to become the most disabling symptoms. Compromised cognitive functioning in children who later develop schizophrenia as compared with their peers is already present at age 4^116^ and may well be impaired from the very beginning. In addition, there is evidence for a deterioration in cognitive and social functioning in early adolescence.^21^ While interventions to relieve psychotic symptoms in the UHR period or during psychotic episodes have only limited impact on long-term functional outcome, preventing cognitive and social decline during childhood or early adolescence may have substantial impact on long-term functioning. In this perspective, we propose that the term premorbid may no longer be accurate when used in schizophrenia as it erroneously suggests that the disease process starts with either the prodromal stage or the onset of the first psychotic symptoms.

Several interventions that are well-tolerated can potentially prevent severe cognitive dysfunction when applied early in the course of the disorder. Such interventions may aim to avoid or mitigate disadvantageous circumstances, such as bullying, social exclusion, or drug use in children at increased risk. Other strategies include the optimization of brain maturation or resilience with interventions to improve the redox balance or immune status of the brain. Since abnormalities in the development and maturation of NMDA and GABA receptors may play an important role in the decline of cognition during childhood and adolescence, this also provides a potential mechanistic background for preventive interventions.

Given the population rate of schizophrenia of about 1%, unfeasibly large samples would be required to provide sufficient power for the study of the efficacy of the interventions discussed here. However, we can now identify a substantial number of selected risk populations in childhood on the basis of genetic or clinical characteristics, such as those with first-degree relatives with schizophrenia, with known genome abnormalities, or children experiencing one or more psychotic symptoms. These early identifiable risk groups provide a rational starting point to examine the efficacy of existing and promising early interventions, with regard to the prevention of social and cognitive deficits in schizophrenia.

Moreover, it is plausible that in the near future neuroimaging,^117^ electroencephalogram, or blood-based biomarkers can be used to further improve the selection of individuals. For instance, reduced P300 amplitude may already be present during childhood and could thus be used to improve selection.^118^ Another example is the measurement of increased inflammatory parameters (e.g., C-reactive protein, IL1β, IL-6, or other parameters) in children with psychotic symptoms to prioritize those who may benefit most from supplements such as NAC or PUFA.

Although the field generally agrees that early intervention is one of the most important goals in schizophrenia research, only few studies directly test efficacy of such intervention strategies. A possible explanation for this paucity is that most risk groups have rather low conversion rates, with only 10–25% of youngsters developing the disorder. This may indeed be a reason for not testing interventions with moderate to severe side effects. However, most preventive interventions described in this manuscript have very mild side-effect profiles, and even reach beyond *non nocere* as they are beneficial even to those who will not develop schizophrenia.

We now have a basic understanding of how to act early and therefore it is time to proceed. Randomized clinical trials are urgently needed to provide a scientifically sound basis for the use of early-intervention strategies in clinical practice. Moreover, we need to evaluate the cost-effectiveness of these interventions. The high-risk groups discussed in this review provide an appealing and strategic starting point. The required trials will be costly and challenging but it is time they are initiated, given their high potential to uncover novel possibilities for preventing or at least mitigating the course of schizophrenia, perhaps even in the near future.
